# Fetoscopic Tracheal Occlusion for Isolated Severe Left Diaphragmatic Hernia: A Systematic Review and Meta-Analysis

**DOI:** 10.3390/jcm13123572

**Published:** 2024-06-18

**Authors:** Henrique Provinciatto, Maria Esther Barbalho, Edward Araujo Júnior, Rogelio Cruz-Martínez, Pankaj Agrawal, Gabriele Tonni, Rodrigo Ruano

**Affiliations:** 1 Department of Medicine, Barao de Maua University Center, Ribeirao Preto 14090-062, SP, Brazil; henriquegprovinciatto@gmail.com; 2 Department of Medicine, Potiguar University, Natal 59056-000, RN, Brazil; barbalhoesther52@gmail.com; 3 Department of Obstetrics, Paulista School of Medicine, Federal University of Sao Paulo, São Paulo 04023-062, SP, Brazil; araujojred@terra.com.br; 4 Fetal Medicine Mexico Institute, Guadalajara 45606, Jalisco, Mexico; rcruz@medicinafetalmexico.com; 5 Division of Neonatology, Department of Pediatrics, University of Miami Miller School of Medicine, Miami, FL 33136, USA; pagrawal@miami.edu; 6 Department of Obstetrics and Neonatology, and, Researcher, Istituto di Ricovero e Cura a Carattere Scientifico (IRCCS), Azienda USL Reggio Emilia, 42122 Reggio Emilia, Italy; gabriele.tonni@ausl.re.it; 7 Division of Maternal-Fetal Medicine, Department of Obstetrics, Gynecology and Reproductive Sciences, University of Miami Miller School of Medicine, 1120 NW 14th Street, Suite # 1152, Miami, FL 33136, USA

**Keywords:** fetal endoscopic tracheal occlusion, congenital diaphragmatic hernia, left diaphragmatic hernia, pulmonary hypoplasia, fetal lung, fetal surgery, fetoscopy

## Abstract

**Background:** We aimed to conduct a systematic review and meta-analysis to evaluate the fetoscopic tracheal occlusion in patients with isolated severe and left-sided diaphragmatic hernia. **Methods:** Cochrane Library, Embase, and PubMed (Medline) databases were searched from inception to February 2024 with no filters or language restrictions. We included studies evaluating the outcomes of fetoscopic intervention compared to expectant management among patients with severe congenital diaphragmatic hernia exclusively on the left side. A random-effects pairwise meta-analysis was performed using RStudio version 4.3.1. **Results:** In this study, we included 540 patients from three randomized trials and five cohorts. We found an increased likelihood of neonatal survival associated with fetoscopic tracheal occlusion (Odds Ratio, 5.07; 95% Confidence Intervals, 1.91 to 13.44; *p* < 0.01) across general and subgroup analyses. Nevertheless, there were higher rates of preterm birth (OR, 5.62; 95% CI, 3.47–9.11; *p* < 0.01) and preterm premature rupture of membranes (OR, 7.13; 95% CI, 3.76–13.54; *p* < 0.01) in fetal endoscopic tracheal occlusion group compared to the expectant management. **Conclusions:** Our systematic review and meta-analysis demonstrated the benefit of fetoscopic tracheal occlusion in improving neonatal and six-month postnatal survival in fetuses with severe left-sided CDH. Further studies are still necessary to evaluate the efficacy of tracheal occlusion for isolated right-sided CDH, as well as the optimal timing to perform the intervention.

## 1. Introduction

Congenital diaphragmatic hernia (CDH) is a congenital condition where part of the diaphragm does not close enough, allowing for herniation of abdominal organs into the fetal chest thus leading to compression of the fetal lungs and heart [1]. Approximately 1 in 4000 pregnancies produces a fetus affected by congenital diaphragmatic hernia [2]. This condition compromises airway and pulmonary vascular development through an intrathoracic herniation of the abdominal viscera, which explains the high rates of postnatal mortality related to pulmonary hypoplasia and pulmonary arterial hypertension [3].

The advancement of perinatology has enabled the opportunity for an accurate prenatal diagnosis, planning of the delivery in a specialized tertiary center, in-utero fetal transfer, and the best postnatal management with the possibility of extracorporeal membrane oxygenation (ECMO) support and surgical repair for these newborns [4]. However, even with all the advancements in medicine, postnatal mortality and morbidity in newborns and infants with severe pulmonary hypoplasia and pulmonary arterial hypertension remain high [5,6].

Therefore, fetal endoscopic tracheal occlusion (FETO) has been proposed for those fetuses with severe CDH [7]. FETO is an intrauterine intervention characterized by the placement of a detachable balloon inside the fetal trachea to promote lung growth in fetuses with CDH [8]. Prenatal pulmonary measurements play a pivotal role as the primary predictors in determining the severity of CDH [9]. Many studies have focused on evaluating different ultrasound and magnetic resonance imaging parameters to classify the severity of CDH [10,11,12,13]. Thus, based on a meta-analysis, severe left-sided CDH can be considered when the observed-to-expected fetal lung area-to-head circumference (o/e-LHR) is below 25%, the observed-to-expected total fetal lung volume (o/e-TFLV) is below 35%, or the lung-to-head ratio (LHR) is below one [14]. Nevertheless, the Tracheal Occlusion to Accelerate Lung Growth (TOTAL) trials attempted to unify the severity criteria of CDH through the o/e-LHR [15,16], which has been supported in accordance with a comprehensive cohort [17,18].

Recently, two randomized controlled trials (RCTs) suggested the potential benefits of FETO in improving postnatal survival for severe CDH [15,19]. Such results have also been confirmed in two meta-analyses [20,21]. In addition, FETO has been associated with perinatal complications such as preterm delivery and premature rupture of the membranes [22,23]. However, such studies used different inclusion criteria to define severe CDH and had included both right- and left-sided CDH cases [15,19].

Therefore, in this systematic review and meta-analysis, we propose to evaluate the safety and efficacy of FETO for isolated severe left-sided CDH in comparison with the expectant management. We also aim to explore these results using a single definition of severity based on the o/e-LHR, and a subgroup analysis addressing the possible heterogeneity according to the study designs. Furthermore, the certainty of the evidence is assessed through a trial sequential analysis (TSA).

## 2. Materials and Methods

In this meta-analysis, we selected studies fulfilling all the predefined eligibility criteria as follows: (1) RCTs and cohorts; (2) that compare FETO with prenatal expectant management; (3) in fetuses with severe; (4) left-side CDH. Studies were excluded in the following situations: (1) overlapping populations, defined as studies that recruited the same patients from the same institutions during overlapping periods; (2) articles published solely as conference abstracts; (3) no outcomes of interest; (4) inclusion of only patients with right-sided CDH and (5) repetition of the cohort of fetuses. In instances where fetuses with both CDH on the right and left side were included, we contacted the study authors to acquire the individual patient data (IPD). Those studies failing to provide IPD were excluded from this meta-analysis. CDH severity was defined according to the institutional criteria based on either the o/e-LHR (≤25%), LHR (<1), or o/e-TFLV (≤35%). We also confirmed that two studies from the same group did not report on the same patients; therefore, confirming that there were no overlapping populations [19,24]. Our study was prospectively registered on the Prospective Register of Systematic Reviews (PROSPERO; CRD42024525796) on 30 March 2024.

We independently searched the Pubmed, Embase, and Cochrane Central databases covering the period from inception to February 2024. No filters or language limitations were employed.

Our search strategy employed the following terms, and their respective Medical Subjective Headings of each database, interconnected by Boolean operators: “congenital diaphragmatic hernia”, congenital, “diaphragmatic hernia”, left, severe, and “fetoscopic tracheal occlusion”. We have provided the full search strategy for each database on Appendix A.

We utilized Rayyan software to record all studies exported from the main databases. Two reviewers (H.P., and M.B.), masked from each other’s decisions, independently selected the RCTs based on prespecified eligibility criteria and documented their decisions. After unmasking, the authors compared their selections and a third author (E.A.J.) then addressed any disagreements.

Study design, baseline characteristics, severity criteria, sample size, and summarized number of events from each outcome were independently collected and reviewed using Microsoft Excel (Microsoft Corp., Redmond, WA, USA). Subsequently, we compared the extracted data and conducted other review sessions in case of eventual discrepancies. Moreover, in cases with obtained IPD, we separated patients by intervention arms, and each event was added to the final data. The main endpoint of interest was neonatal survival. Our secondary outcomes included 6-month postnatal survival, preterm birth before 37, 34, and 32 weeks of pregnancy, preterm premature rupture of membranes (PPROM), and placental abruption. We defined neonatal survival as survival until 28 days of age, while PPROM included instances of membrane rupture before 37 weeks of gestation. All endpoints were considered binary outcomes and were extracted as the number of patients who had each event. We also separated data of neonatal survival according to an o/e-LHR < 25%, aiming to perform our prespecified subgroup analysis of novel classification for severity degree.

Two authors independently evaluated the quality of each study in our meta-analysis through version 2 of the Cochrane Risk of Bias Assessment Tool (ROB-2) for RCTs. Included trials were scored as high, some concerns, or low risk of bias, following the recommendations from Cochrane Collaboration Handbook. For observational studies, we utilized the Risk of Bias in Non-Randomized Studies of Interventions (ROBINS-I) to classify the included cohorts as critical, high, moderate, or low risk of bias.

After completing the quality assessment, both authors compared their results, and resolved any discrepancy by consensus. We planned to conduct sensitivity analyses using the leave-one-out, Baujat, and L’abbé tests to assess our primary outcome of neonatal survival, and we intended to then generate a funnel plot for each outcome to explore the possibility of publication bias.

Our meta-analysis utilized the random-effects and restricted maximum-likelihood estimators to address potential disparities across the included trials. In addition, computation of Odds ratio (OR) for dichotomous outcomes was performed with the Inverse Variance method. At a significance level of 0.05, we identified statistical differences between the intervention and control groups when the *p*-value fell below this threshold and the CI did not encompass the null effect line. We employed Cochran’s Q test and I2 statistics to estimate heterogeneity. I2 values of <40%, 40–75%, and >75% were classified as representing low, moderate, and high heterogeneity, respectively.

Moreover, we conducted subgroup analyses for our main outcome according to study design and o/e-LHR. We also performed a funnel plot and Egger’s regression test as needed to investigate heterogeneity between the study-specific estimates. Our meta-analysis was conducted using the meta-package for RStudio version 4.3.1 (R Foundation for Statistical Computing, Vienna, Austria).

Furthermore, we utilized TSA to evaluate if the collective evidence had sufficient power. Our study established an intervention effect of a 20% reduction in OR for the assessed outcomes. Specifically, we focused our analysis on the subgroup of studies with o/e-LHR lower than 25% for postnatal survival at six months of follow-up. We conducted a two-sided test with a 5% type I error rate and aimed for a 20% type II error rate (80% power). For comparing the intervention and control groups, we constructed both conventional boundaries (at 5% α) and trial sequential monitoring boundaries. In TSA, we employed the random-effects model and generated a cumulative sequential Z-score curve. Additionally, using TSA version 9.5.10 from Copenhagen, Denmark, we calculated the diversity-adjusted required information size.

## 3. Results

### 3.1. Study Selection

**Figure 1:**
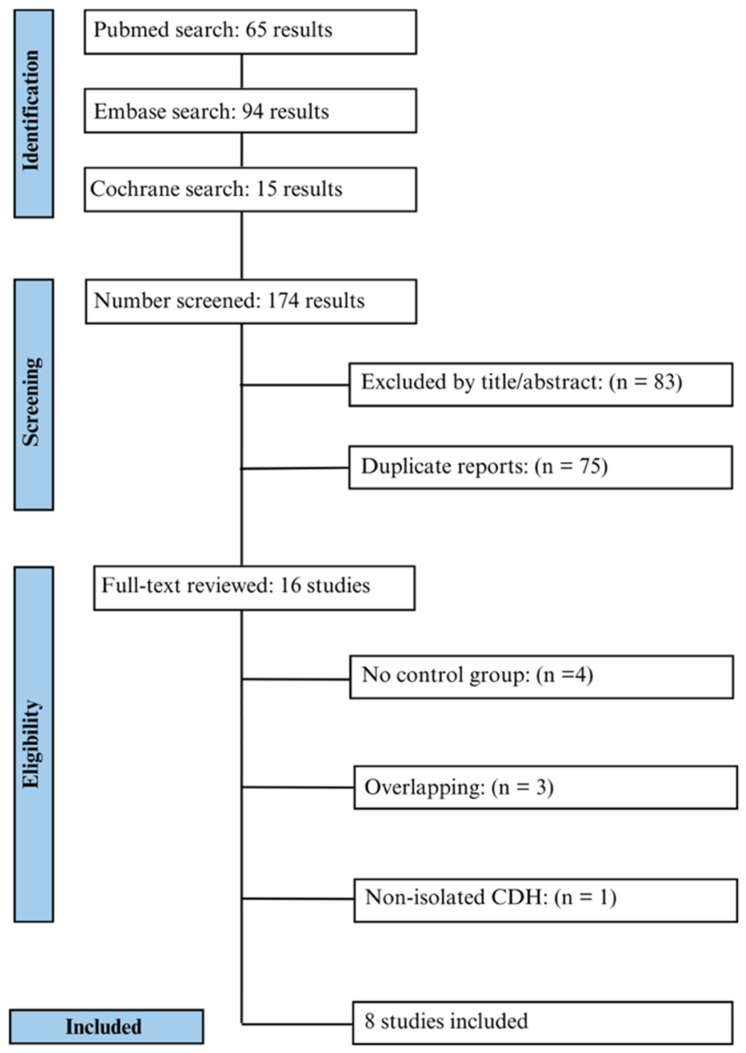
PRISMA flow diagram.

Following our initial search, which produced 174 records, we eliminated 75 duplicates, resulting in 99 studies for title and abstract screening. From these, we thoroughly assessed 16 studies based on predefined eligibility criteria. Thereafter, our selection included three RCTs and five cohorts. Ultimately, the selected studies comprised 540 patients, of whom 238 (44.1%) had undergone FETO. Figure 1 summarizes the Preferred Reporting Items for Systematic Review and Meta-Analyses (PRISMA) flow diagram.

### 3.2. Study Characteristics

Individual characteristics of the included studies are available in Table 1 [15,19,23,24,25,26,27,28]. Each RCT defined a different set of inclusion criteria for enrolling the participants. While two RCT studies based their selection on an LHR of below 1.0 [19,28], the parallel TOTAL trial selected patients according to an o/e-LHR of less than 25% [15]. Concerning the side of isolated diaphragmatic hernia, two RCTs included only fetuses with left-sided CDH [15,28]. The other study enrolled patients with either left- or right-sided CDH [19]. Nevertheless, the IPD of those with CDH solely on the left side were provided by contacting the author of the study. Additionally, there was no uniformity regarding the time period to perform FETO, with the range varying from 22 to 30 weeks. Similarly, the time period for balloon removal was also different between the studies.

Among the included cohorts, two studies used an LHR of below 1.0 to define severe cases [24,27], two cohorts employed o/e-LHR equal to or less than 25% [23,25], while one study assessed severity based on o/e-TFLV < 35% [26]. Four studies exclusively included patients with left-sided CDH, and one cohort also incorporated data from patients with right-sided CDH [24]. In this context, the IPD of those diagnosed with CDH on the left side were supplied by the corresponding author. In line with RCTs, the timing of tracheal balloon removal also differed, and we found a variability in gestational age as the eligibility criteria to perform FETO within the selected cohorts, which ranged from 27 to less than 32 weeks.

### 3.3. Risk of Bias of Included Studies

Figure S1 summarizes the individual evaluation of each included study in the meta-analysis utilizing the RoB-2 and ROBINS-I quality assessment tools. While all the RCTs were assessed as having an overall low risk of bias, the selected cohorts varied in bias, ranging from low to critical risk. We downgraded the retrospective studies due to their potential for confounding factors, particularly in cases where there was a significant dissimilarity between the baseline characteristics of the intervention and control groups. Moreover, we attributed critical concerns of bias in cases where the methodological designs may have affected the results.

### 3.4. Synthesis of Results

**Figure 2:**
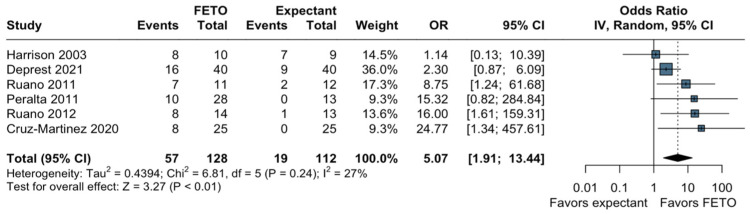
Forest plot of neonatal survival with fetoscopic intervention and expectant management. Harrison 2003 [28]; Deprest 2021 [15]; Ruano 2011 [24]; Peralta 2011 [27]; Ruano 2012 [19]; Cruz-Martinez 2020 [25].

**Figure 3:**
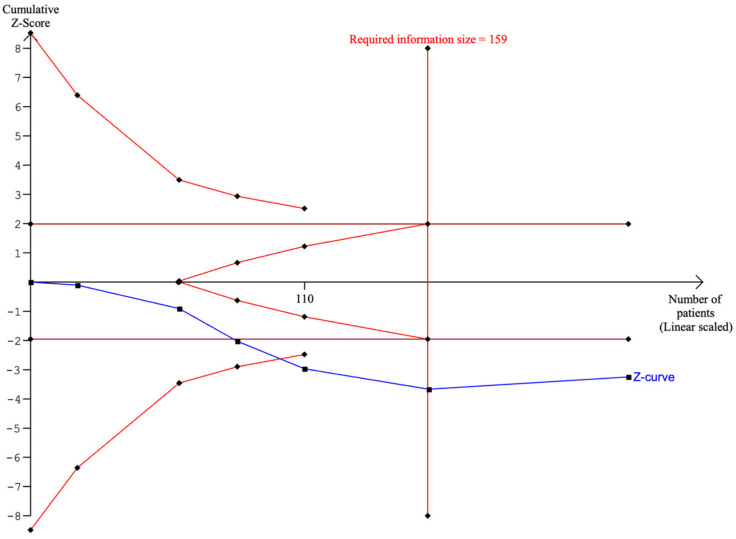
Trial sequential analysis of neonatal survival with fetoscopic tracheal occlusion and expectant management.

Of the eight studies, six articles reported our primary outcome of neonatal survival (until 28 days of age). The pooled analysis demonstrated a statistically significant association between FETO and neonatal survival compared to the expectant management when considering LHR, o/e-LHR or o/e-TFLV (OR, 5.07; 95% CI, 1.91–13.44; *p* < 0.01; I2 = 27%; Figure 2). We also found a statistically significant increase in neonatal survival associated with FETO in comparison with the group managed expectantly during pregnancy (OR, 4.94; 95% CI, 1.43–17.10; *p* = 0.01; I2 = 38%; Figure S2) when performing our prespecified subgroup analysis of o/e-LHR < 25%, and the estimated heterogeneity remained low. There was no significant interaction concerning our subgroup analysis between the randomized and non-randomized studies (*p* = 0.08; Figure S3). Regarding the TSA for neonatal survival, the cumulative sequential z-curve crossed the conventional boundary for benefit, indicating the beneficial effect of FETO. Furthermore, the curve also surpassed the RIS, providing an accurate certainty of benefit (Figure 3).

Four studies (two RCTs and two cohorts) reported postnatal survival of 6 months, and the pooled analysis resulted in a significant association with FETO compared to expectant management (OR, 3.38; 95% CI, 1.46–7.80; *p* < 0.01; I2 = 36%; Figure S4).

**Figure 4:**
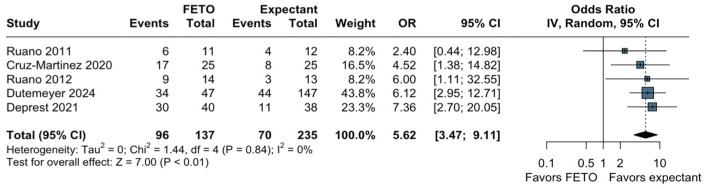
Forest plot of preterm birth before 37 weeks. Ruano 2011 [24]; Cruz-Martinez 2020 [25]; Ruano 2012 [19]; Dutemeyer 2024 [26]; Deprest 2021 [15].

Five studies reported preterm birth before 37 weeks, and we found an increased risk of this adverse outcome in the group treated with fetoscopy (OR, 5.62; 95% CI, 3.47–9.11; *p* < 0.01; I2 = 0; Figure 4) compared to expectant management in our pooled analysis. Similarly, our analyses encompassing three of the studies demonstrated an association between FETO and preterm delivery before 34 (OR, 7.51; 95% CI, 1.59–35.41; *p* = 0.01; Figure S5) and 32 weeks of pregnancy (OR, 7.87; 95% CI, 1.59–38.95; *p* = 0.01; Figure S6), respectively.

**Figure 5:**
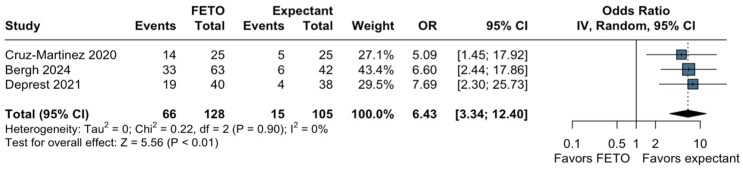
Forest plot of preterm premature rupture of membranes. Cruz-Martinez 2020 [25]; Bergh 2024 [23]; Deprest 2021 [15].

In a pooled analysis of three studies for the risk of PPROM, we observed a statistically higher incidence of PPROM in the group of FETO (OR, 6.43; 95% CI, 3.34–12.40; I2 = 0%; *p* < 0.01; Figure 5) compared to the expectant group. Nevertheless, there was no association between fetoscopic intervention and placental abruption (OR, 1.14; 95% CI, 0.14–9.62; *p* = 0.90; I2 = 0%; Figure S7). Three studies reported the need for ECMO, and the pooled analysis revealed a notable decrease in the likelihood of fetuses requiring ECMO (OR, 0.15; 95% CI, 0.03–0.74; *p* = 0.02; Figure S8).

There was no indication of publication bias based on the examination of funnel plots. However, it is important to highlight that the maximum number of studies included in a single pooled analysis of our review did not exceed the recommended threshold of ten studies to perform a proper assessment or the Egger test (Figures S9–S16).

## 4. Discussion

In this systematic review and meta-analysis, we included 540 patients from three RCTs and five cohorts comparing FETO and prenatal expectant management in fetuses with isolated CDH on the left side. Our main findings are the following: (1) there is a significantly higher survival rate among fetuses with isolated severe left-sided CDH treated with FETO compared to those managed expectantly during pregnancy; (2) the improvement in survival rate remained significant with FETO among cases with o/e-LHR < 25% (recent proposed inclusion criterion for severe left-sided CDH); (3) there is no interaction between the study designs and the results found in our meta-analysis (randomized controlled studies versus non-randomized studies); (4) FETO is associated with a reduced chance of needing ECMO; (6) FETO is associated with a statistically increased risk of complications including preterm birth before 37 weeks’ gestation and 32 weeks’ pregnancy; (7) there is a higher risk of PPROM when performing fetoscopic intervention in comparison with expectant management; and (8) there is no association between FETO and placental abruption.

Contrary to the previously published meta-analysis that included RCTs with mixed left- and right-sided CDHs [20], our meta-analysis employed the IPD restricted to patients only with left-sided and severe CDH. This is important when providers counsel their patients because the survival rates with FETO compared to expectant management are different between right- and left-sided CDHs; the survival rates for right-sided CDHs and left-sided CDHs are also different [19]. Therefore, further studies are still necessary to determine the prenatal predictor factors in right-sided CDHs, as well as the efficacy of FETO and postnatal outcomes in this cohort of fetuses with right-sided CDHs [29].

In addition, it is important to compare studies that used similar inclusion criteria for FETO. In our study, we prespecified the o/e-LHR below 25% as a subgroup analysis, aiming to clinically compare the same population of fetuses and explore the heterogeneity across the general analysis. In both analyses, our study demonstrates that FETO improves neonatal survival for fetuses with left-sided CDH within the group with severe lung hypoplasia.

Regarding the complications associated with FETO, our study confirms a higher risk of preterm delivery (OR, 5.64; 95% CI, 3.54–9.00; *p* < 0.01), and premature rupture of the membranes (OR, 7.13; 95% CI, 3.76–13.54; *p* < 0.01).

There is also controversy regarding the efficacy of FETO for moderate left-sided CDH. Only one RCT and one cohort have evaluated the efficacy of FETO for fetuses with left-sided CDHs and moderate lung hypoplasia (o/e-LHR between 25% and 35%) and both studies were not able to show a significant association between FETO and neonatal survival [16]. However, neonatal morbidity was significantly lower in the group of FETO. In addition, in a recent reanalysis of such RCT, the authors were able to suggest that FETO may improve survival rate in moderate left-sided CDHs in the subgroup of cases treated before 30 weeks [30]. This conclusion was derived from a logistic regression with a penalized maximum likelihood estimation that comprised 287 patients, of whom 196 had moderate lung hypoplasia. In keeping with this contention, Ruano et al. [31] suggested that early FETO below 26 weeks’ gestation may increase the impact of FETO on improving neonatal survival. The study compared data of eight fetuses who underwent early FETO, along with ten patients managed expectantly and nine that submitted to standard tracheal occlusion. In the early FETO group, 62.5% of patients survived, whereas only 11.1% and 0% of fetuses survived in the standard and control groups, respectively. Based on this analysis, early intervention was associated with significantly higher postnatal survival rates and significantly stronger pulmonary response in fetuses with extremely severe CDHs when compared to ‘classical FETO’ performed at 26–30 weeks’ gestation [31]. Thus, further RCTs evaluating the efficacy of FETO for moderate CDH at earlier gestational ages for fetal intervention are still required to address this issue.

Another aspect to be considered is the application of the RCT results to different populations. The North American Fetal Therapy Network (NAFTnet) FETO Consortium has recently published the experience of different centers in the United States which have failed to demonstrate a significant improvement in postnatal survival even in fetuses with severe conditions [23]. Concerning the six-month survival rate, FETO did not exhibit a significant difference when compared with expectant management (69.8% vs. 58.1). However, the NAFTnet FETO Consortium suggests that FETO may reduce pulmonary morbidity by decreasing the need for ECMO, duration in the NICU, and other pulmonary complications [23]. Similarly, a retrospective multicenter cohort comparing FETO with expectant management found a higher survival rate at discharge and two years of follow-up in the expectant group [26]. The authors included 194 patients with left-sided CDH from four European centers. Of these, 47 (24.2%) fetuses underwent tracheal occlusion, and the remaining patients were expectantly followed. Despite using a different timing of follow-up in their report in comparison with the other studies in our review, Dutemeyer et al. [26] found that cases not treated with FETO were more likely to be alive at two years of age (OR, 3.61; 95% CI, 1.84–7.23; *p* < 0.01) [26]. Nonetheless, this study utilized FETO data from three centers and compared it with expectant management data from the highest specialized center.

It is possible that the different results from these two studies compared to the others in the present meta-analysis regarding the impact of FETO in the postnatal survival rates may be related to the use of ECMO and the experience of the center in the postnatal management of infants with CDH. Some studies have suggested that ECMO may improve survival, although this seems to be still controversial [32]. Other studies have even shown that FETO may reduce the need for ECMO in newborns with severe left-sided CDH [33]. Hayakawa et al. [34] suggested that the experience of the center in treating newborns with CDH may impact considerably the outcomes. In our present study, despite including studies from different parts of the world, all these studies were conducted in centers with high levels of experience in treating postnatally babies with CDH. We believe that further studies are still necessary to evaluate these aspects in babies treated with FETO.

Another point that needs further investigation is the long-term outcomes after FETO in comparison with those patients who underwent expectant management. The RCTs and the other non-randomized trials focus on postnatal outcomes after 6 months of life of survival to discharge [15,19,23,24,25,26,27,28]. Further studies are necessary to evaluate the long-term outcomes regarding the mortality, and especially the morbidity, of those infants who have undergone FETO [35] to guide the long-term impact factors on counselling and the ethical considerations [36,37]. Moreover, novel approaches may be developed to prevent FETO-related complications and improve neonatal management [38,39,40].

Our study is the first meta-analysis to fully include available data addressing FETO in comparison of expectant management for left-sided CDH. This approach was made feasible by integrating IPD from two out of the eight studies documented in the literature, facilitating a more standardized analysis of pooled data. Moreover, the subgroup analyses based on study design and o/e-LHR in association with the TSA added a more accurate certainty of evidence regarding the potential benefit of FETO for this population.

It is important to consider the limitations of both studies. The longitudinal design may have introduced potential confounding factors, in addition to the large variation in the severity and laterality of CDH, the period to perform FETO, and an inherent learning curve from each center.

The present systematic review and meta-analysis study has the following limitations: (1) Each RCT has methodological particularities, such as different recruiting periods to perform FETO, and distinct timings of balloon removal, which could not be addressed since we used summarized data from two of the three trials; (2) there were a limited number of studies in the whole literature; and (3) the trials were carried out in highly specialized centers and under controlled conditions, which makes it more challenging to extrapolate these results to centers without experience in fetoscopic intervention.

## 5. Conclusions

In conclusion, based on our trial sequential analysis, we were able to confirm that FETO improves the postnatal outcomes in fetuses with severe left-sided CDH when compared to prenatal expectant management. Fetoscopic intervention increased postnatal survival rates, and decreased the need for ECMO where it is available. Nevertheless, this intervention also carries an increased risk of preterm birth and PPROM, but FETO is not associated with increased risk of placental abruption. Further studies are still warranted to evaluate the effectiveness and impact of FETO in moderate left-sided CDH and in severe right-sided CDH, as well the best timing for tracheal occlusion in those populations.

## Figures and Tables

**Table 1:** Individual studies characteristics.

Study	Country	Sample Size (FETO versus Expectant)	Gestational Age	Severity Criteria	Liver Herniation	Outcomes
Bergh 2024 [23]	Canada, and USA	63 versus 43 patients	27–29 weeks	o/e-LHR < 25%	Both	6-month survival, and PPROM
Cruz-Martinez 2020 [25]	Mexico	25 versus 25 patients	<32 weeks	o/e-LHR ≤ 25%	Only herniated	Neonatal survival, and PPROM
Deprest 2021 [15]	Canada, USA, Belgium, the United Kingdom, France, Spain, Canada, Italy, Japan, the United States, Germany, the Netherlands, Switzerland, and Poland	40 versus 40 patients	27–29 weeks	o/e-LHR < 25%	Both	6-month survival, neonatal survival, preterm birth < 37, <34, and <32 weeks, PPROM, placental abruption, and ECMO
Dutemeyer 2024 [26]	Belgium, France, Germany, and Italy	47 versus 147 patients	27–29 weeks	o/e-TFLV ≤ 35%	Only herniated	Preterm birth < 37 weeks, and ECMO
Harrison 2003 [28]	USA	10 versus 9 patients	22–27 weeks	LHR < 1.06	Only herniated	Neonatal survival
Peralta 2011 [27]	Brazil	28 versus 13 patients	<27 weeks	LHR < 1	Only herniated	Neonatal survival, and placental abruption
Ruano 2011 [24]	Brazil	11 versus 12 patients	27–29 weeks	LHR < 1.0, o/e-LHR < 25%	Only herniated	6-month survival, neonatal survival, preterm birth < 37, <34, and <32 weeks
Ruano 2012 [19]	Brazil	14 versus 13 patients	26–29 weeks	LHR < 1.0, o/e-LHR < 25%	Only herniated	6-month survival, neonatal survival, preterm birth < 37, <34, and <32 weeks

ECMO: extracorporeal membrane oxygenation; LHR: lung-to-head ratio; o/e-LHR: observed-to-expected lung-to-head ratio; o/e-TFLV: observed-to-expected total fetal lung volume; PPROM: preterm premature rupture of membranes; UK: United Kingdom; and USA: United States of America.

## Data Availability

Data supporting reported results are available among included studies. Individual patient data may be available upon request and agreement with the original study’s authors.

## Supplementary Materials

The following supporting information can be downloaded at: https://www.mdpi.com/article/10.3390/jcm13123572/s1, Figure S1: Risk of bias of included studies, Figure S2: Forest plot of neonatal survival based on observed-to-expected lung-to-head ratio, Figure S3: Neonatal survival based on study design, Figure S4: Forest plot of 6-month postnatal survival, Figure S5: Forest plot of preterm birth before 34 weeks, Figure S6: Forest plot of preterm birth before 32 weeks, Figure S7: Forest plot of placental abruption, Figure S8: Forest plot of need of extracorporeal membrane oxygenation, Figure S9: Funnel plot of neonatal survival, Figure S10. Funnel plot of 6-month postnatal survival, Figure S11: Funnel plot of preterm birth before 37 weeks, Figure S12: Funnel plot of preterm birth before 34 weeks, Figure S13: Funnel plot of preterm birth before 32 weeks, Figure S14: Funnel plot of preterm premature rupture of membranes, Figure S15: Funnel plot of placental abruption, Figure S16: Funnel plot of need of extracorporeal membrane oxygenation.

## Abbreviations

CI	Confidence Interval
CDH	Congenital diaphragmatic hernia
ECMO	Extracorporeal membrane oxygenation
FETO	Fetoscopic endoluminal tracheal occlusion
IPD	Individual patient data
LHR	Lung-to-head ratio
NAFTNet	North American Fetal Therapy Network
NICU	Neonatal Intensive Care Unit
O/e-LHR	Observed-to-expected lung-to-head ratio
O/e-TFLV	Observed-to-expected total fetal lung volume
PPROM	Preterm premature rupture of membranes
PRISMA	Preferred Reporting Items for Systematic Review and Meta-Analyses
RCT	Randomized Controlled Trial
ROB-2	Version 2 of the Cochrane Risk of Bias Assessment Tool
ROBINS-I	Risk of Bias in Non-Randomized Studies of Interventions
RR	Risk Ratio
TOTAL	Tracheal Occlusion to Accelerate Lung Growth
TSA	Trial sequential analysis

## Appendix A

### Search Strategy

Pubmed
(“Hernias, Diaphragmatic, Congenital”[mh]. OR “congenital diaphragmatic hernia” OR ((congenital[subheading]. OR congenital) AND (“hernia, diaphragmatic”[mh]. OR “diaphragmatic hernia”))) AND (left OR left-sided) AND (severe) AND (fetoscopic OR fetoscopy OR FETO OR “tracheal occlusion”)
Embase
(“congenital diaphragm hernia”/exp OR “congenital diaphragmatic hernia” OR ((congenital/exp OR congenital) AND (“diaphragm hernia” OR “diaphragmatic hernia”))) AND (left OR left-sided) AND (severe) AND (fetoscopic OR fetoscopy OR FETO OR “tracheal occlusion”)
Cochrane Central
(“congenital diaphragmatic hernia” OR ((congenital) AND (“diaphragmatic hernia”))) AND (left OR left-sided) AND (severe) AND (fetoscopic OR fetoscopy OR FETO OR “tracheal occlusion”)
