# Supplementary material for: Fetoscopic Tracheal Occlusion for Isolated Severe Left Diaphragmatic Hernia: A Systematic Review and Meta-Analysis
Source: J Clin Med. 2024 Jun 18;13(12):3572. doi: 10.3390/jcm13123572 (PMC11204948; doi:10.3390/jcm13123572)
Supplement: Supplementary file 1 [file jcm-13-03572-s001.zip › jcm-2916290-supplementary.pdf]

Journal of
Clinical Medicine

MDPI

Supplementary Files

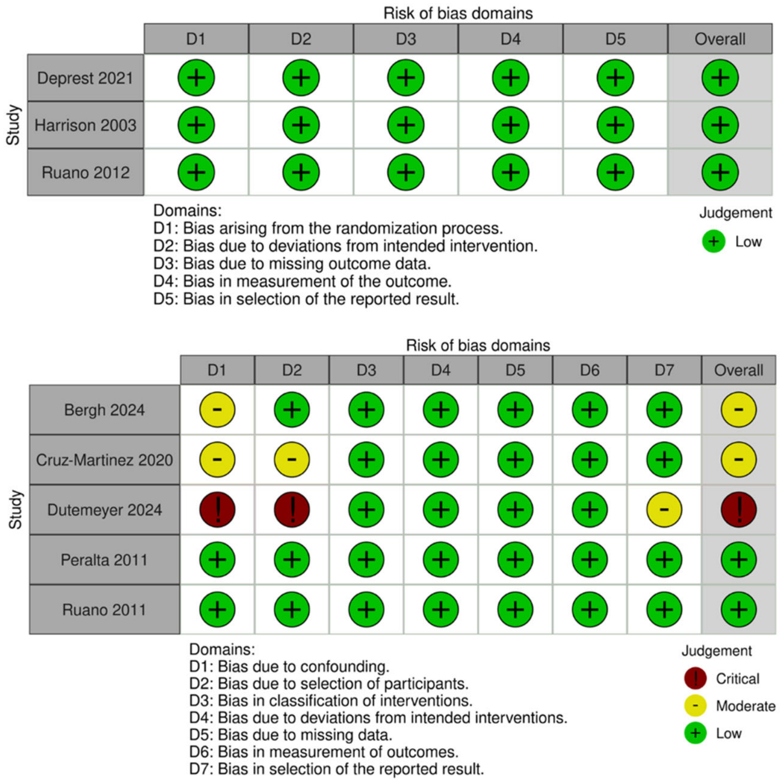

Figure S1. Risk of bias of included studies.

Study	FETO Events	FETO Total	Expectant Events	Expectant Total	Weight	OR	95% CI
Deprest 2021	16	40	9	40	52.3%	2.30	[0.87; 6.09]
Ruano 2011	7	11	2	12	26.5%	8.75	[1.24; 61.68]
Ruano 2012	8	14	1	13	21.2%	16.00	[1.61; 159.31]
**Total (95% CI)**	**31**	**65**	**12**	**65**	**100.0%**	**4.94**	**[1.43; 17.10]**

Heterogeneity: $Tau^2 = 0.5203$; $Chi^2 = 3.22$, df = 2 (P = 0.20); $I^2 = 38\%$
Test for overall effect: Z = 2.52 (P = 0.01)

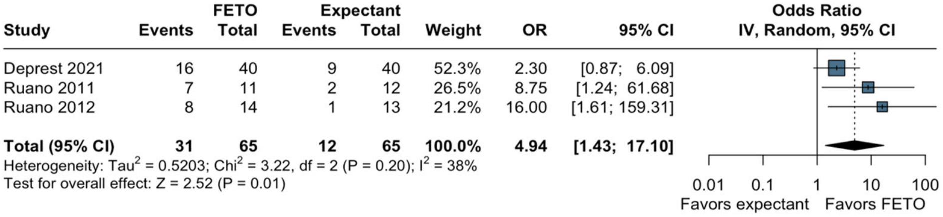

Figure S2. Forest plot of neonatal survival based on observed-to-expected lung-to-head ratio. Deprest 2021 [15]; Ruano 2012 [19]; Ruano 2011 [24].

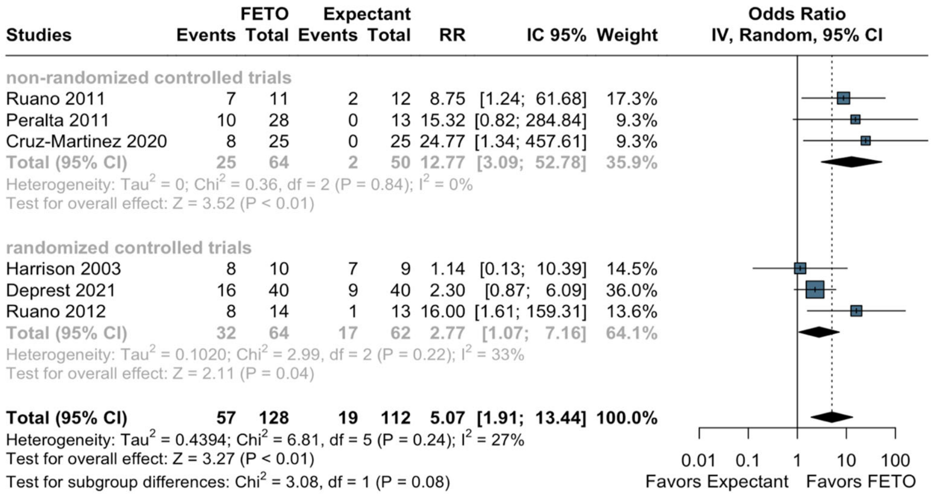

Figure S3. Neonatal survival based on study design. Deprest 2021 [15]; Ruano 2012 [19]; Ruano 2011 [24]; Cruz-Martinez 2020 [25]; Peralta 2011 [27]; Harrison 2003 [28].

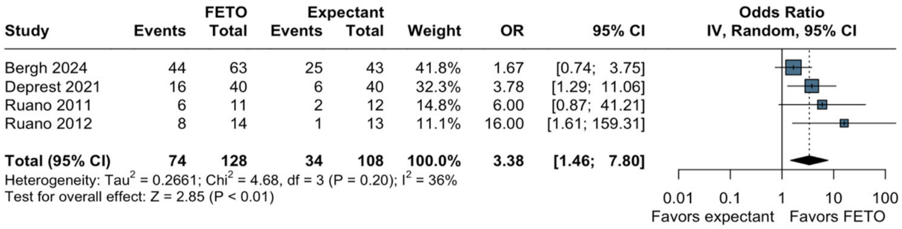

Figure S4. Forest plot of 6-month postnatal survival Deprest 2021 [15]; Ruano 2012 [19]; Bergh 2024 [23]; Ruano 2011 [24].

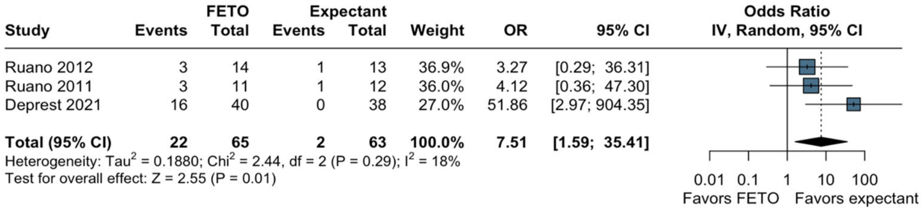

Figure S5. Forest plot of preterm birth before 34 weeks. Deprest 2021 [15]; Ruano 2012 [19]; Ruano 2011 [24].

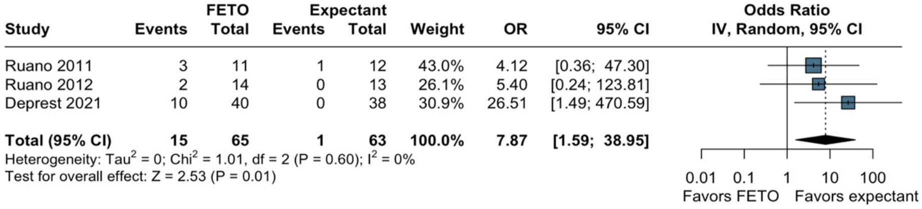

Figure S6. Forest plot of preterm birth before 32 weeks. Deprest 2021 [15]; Ruano 2012 [19]; Ruano 2011 [24].

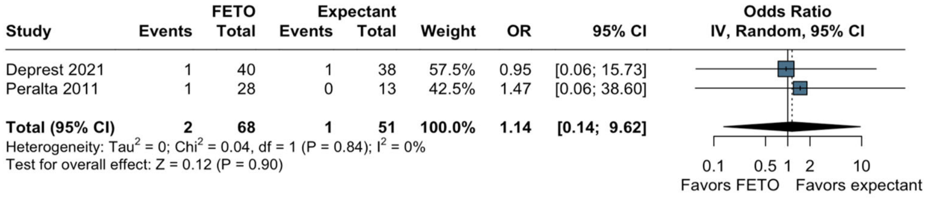

Figure S7. Forest plot of placental abruption. Deprest 2021 [15]; Peralta 2011 [27].

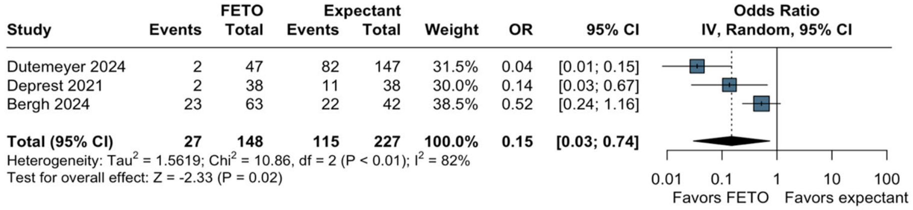

Figure S8. Forest plot of need of extracorporeal membrane oxygenation. Deprest 2021 [15]; Bergh 2024 [23]; Dutemeyer 2024 [26].

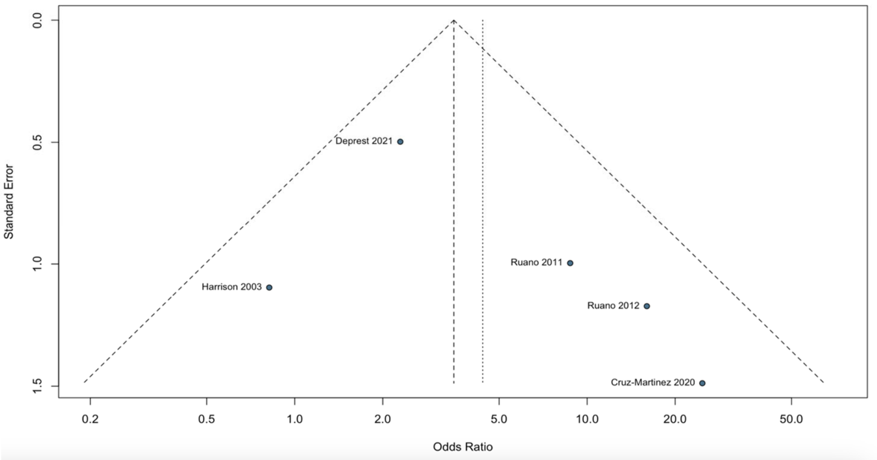

Figure S9. Funnel plot of neonatal survival. Deprest 2021 [15]; Ruano 2012 [19]; Ruano 2011 [24]; Cruz-Martinez 2020 [25]; Harrison 2003 [28].

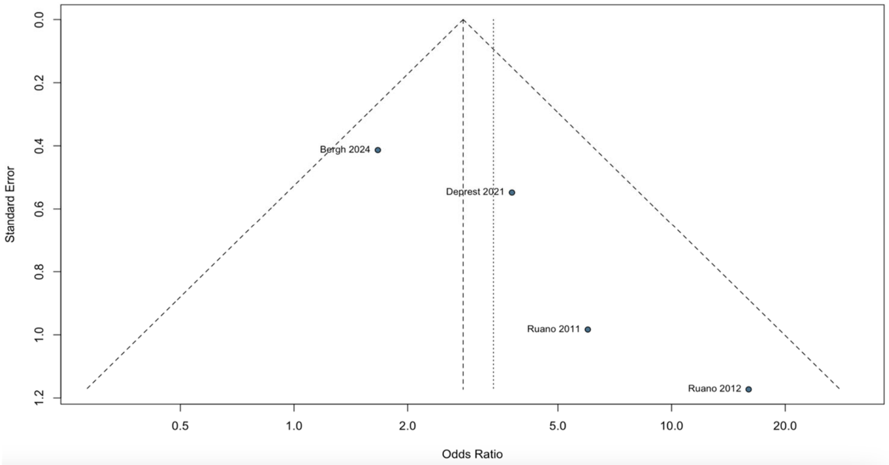

Figure S10. Funnel plot of 6-month postnatal survival. Deprest 2021 [15]; Bergh 2024 [23]; Ruano 2011 [24].

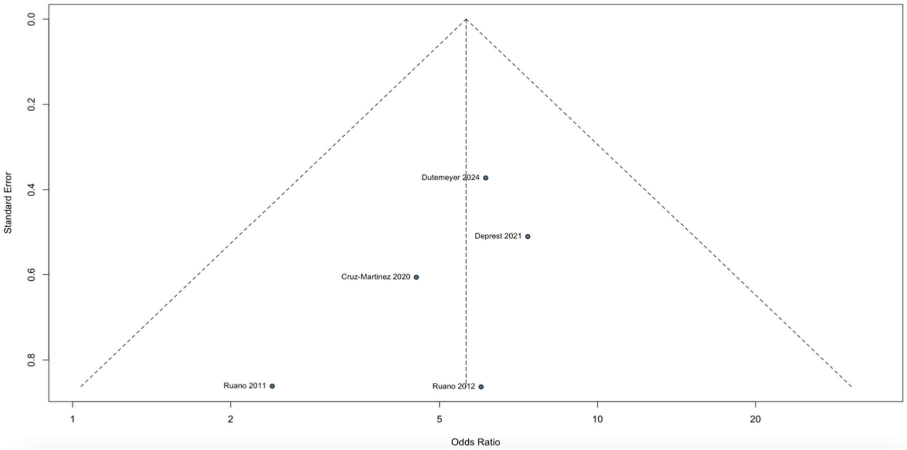

Figure S11. Funnel plot of preterm birth before 37 weeks. Deprest 2021 [15]; Ruano 2012 [19]; Ruano 2011 [24]; Cruz-Martinez 2020 [25]; Dutemeyer 2024 [26].

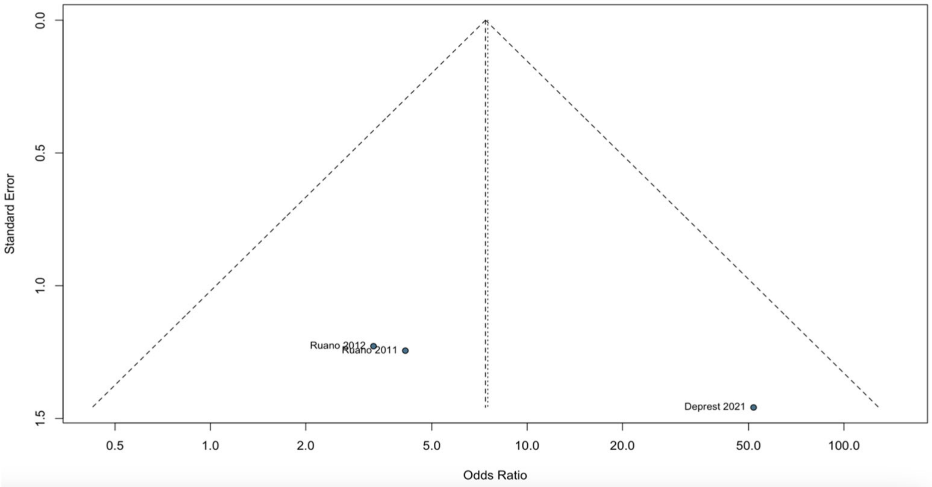

Figure S12. Funnel plot of preterm birth before 34 weeks. Deprest 2021 [15]; Ruano 2012 [19]; Ruano 2011 [24].

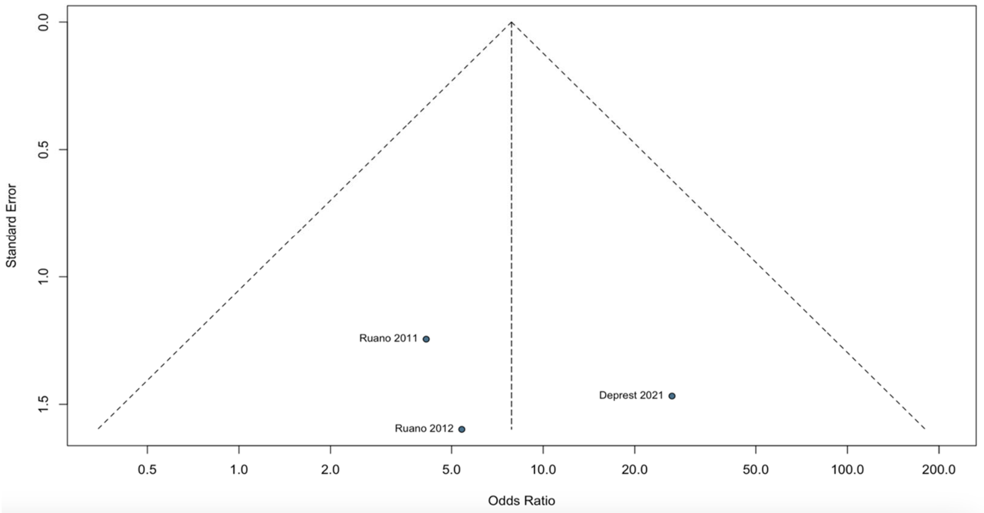

Figure S13. Funnel plot of preterm birth before 32 weeks. Deprest 2021 [15]; Ruano 2012 [19]; Ruano 2011 [24].

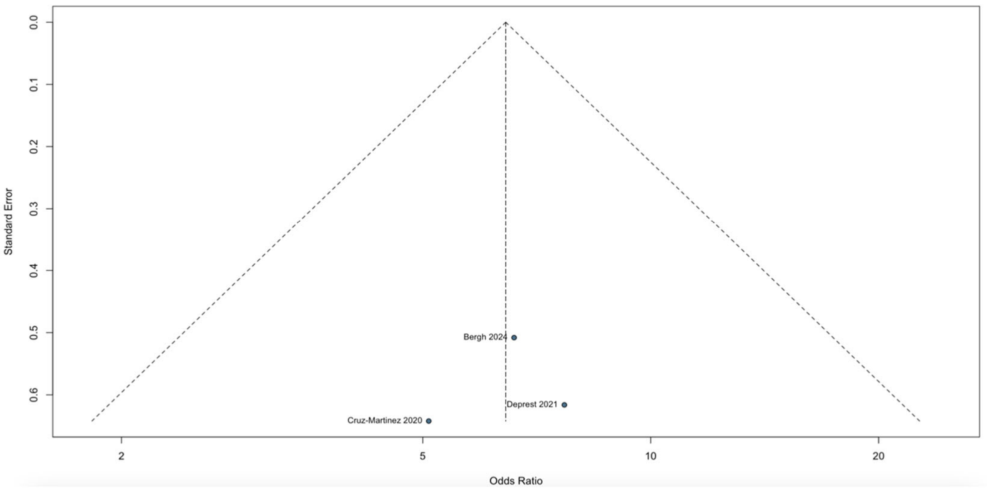

Figure S14. Funnel plot of preterm premature rupture of membranes. Deprest 2021 [15]; Bergh 2024 [23]; Cruz-Martinez 2020 [25].

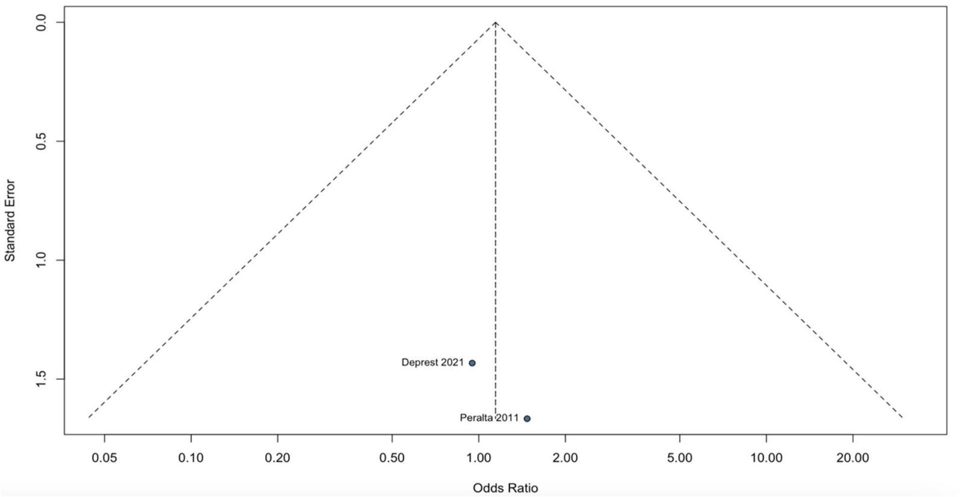

Figure S15. Funnel plot of placental abruption. Deprest 2021 [15]; Peralta 2011 [27].

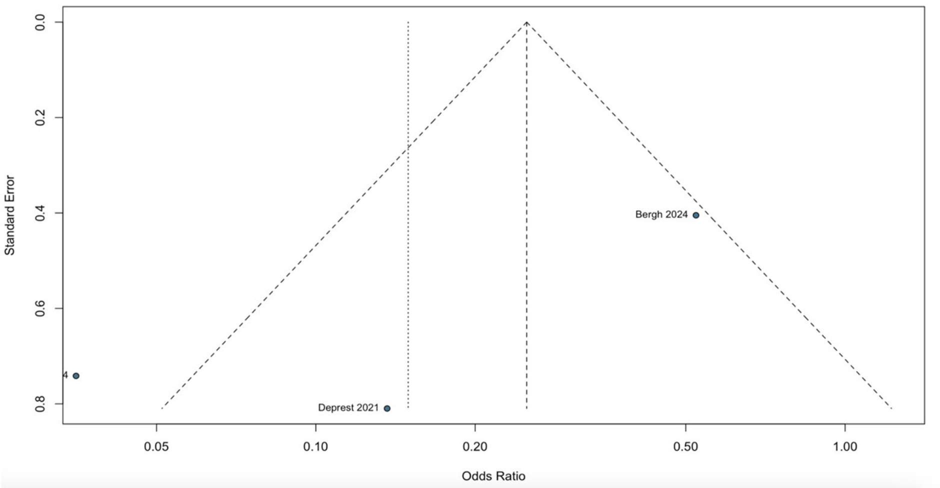

Figure S16. Funnel plot of need of extracorporeal membrane oxygenation. Deprest 2021 [15]; Bergh 2024 [23].
